# Supplementary material for: Optogenetic gamma stimulation rescues memory impairments in an Alzheimer’s disease mouse model
Source: Nat Commun. 2019 Nov 22;10:5322. doi: 10.1038/s41467-019-13260-9 (PMC6876640; doi:10.1038/s41467-019-13260-9)
Supplement: Supplementary file 1 — Supplementary Information [file 41467_2019_13260_MOESM1_ESM.pdf]

Supplementary information

Optogenetic gamma stimulation rescues memory impairments in an Alzheimer's disease mouse model

Etter et al.

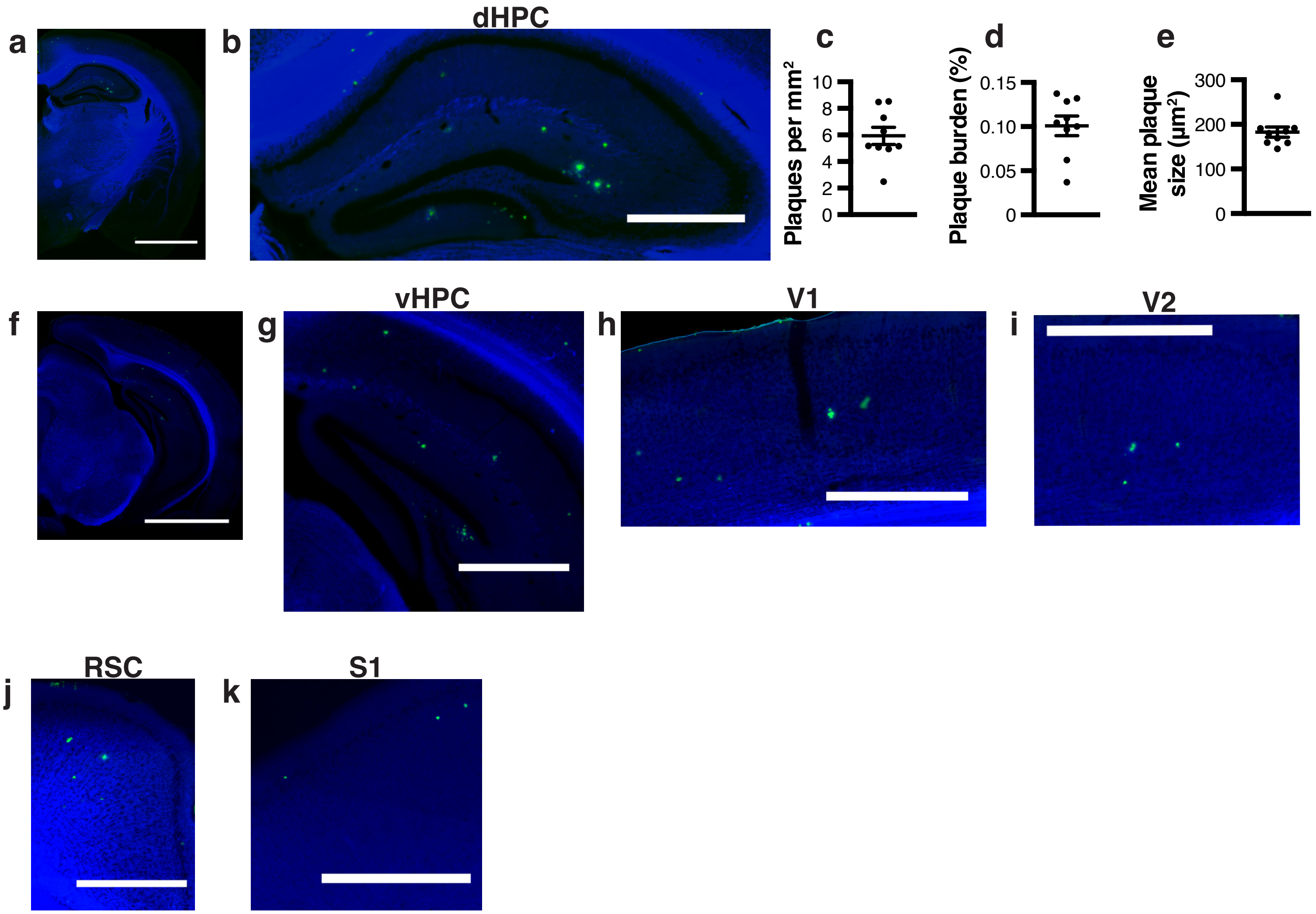

Supplementary figure 1. Thioflavine S staining in 6 month old PVJ20 mice. a, thioflavine S (green) with DAPI counterstaining (blue) in a coronal section containing the dorsal portion of the hippocampus. **b**, magnified view of the dorsal hippocampus. **c-e**, plaque density, plaque burden, and average plaque size respectively for the dorsal hippocampus (n = 9 PVJ20+ mice. Data represented as mean ± SEM). **f**, thioflavine S (green) with DAPI counterstaining (blue) in a coronal section containing the ventral portion of the hippocampus. **g**, magnified view of the ventral hippocampus. **h**, same for visual cortex area 1. **i**, visual cortex area 2. **j**, retrosplenial cortex. **k**, somatosensory area 1. Scale bars in **a**,**f** : 2 mm; b,g: 400 µm; **h**-**k**: 500 µm. Source data are provided as a Source Data file.

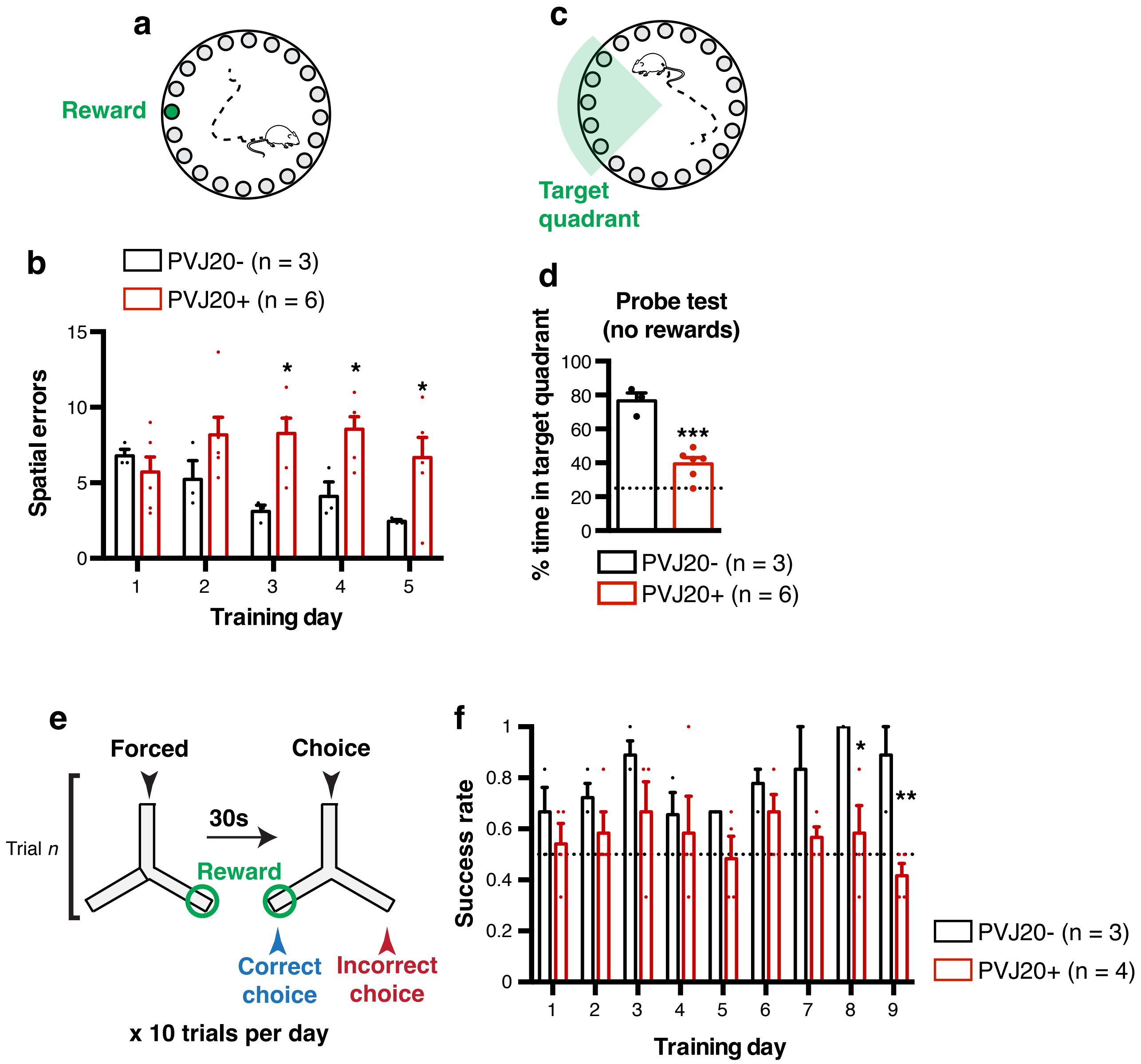

Supplementary figure 2. Behavioral characterization of PVJ20 mice. a, PVJ20 mice were trained on an appetitive version of the Barnes maze where one location is baited. **b**, PVJ20+ mice made significantly more spatial errors (single visit of non-baited location; RM-2ANOVA, $F_{(1,7)} = 32.28$, $p = 0.0274$ for the main effect of genotype; n = 3 PVJ20- mice, n = 6 PVJ20+ mice). **c**, probe test day (no baited location). **d**, PVJ20+ mice explored significantly less the target quadrant (student t-test, $t_7 = 6.084$, $p < 0.005$). **e**, PVJ20 mice were then trained to the delayed non-match to place task in a Y-maze. **f**, PVJ20+ mice never learned to alternate successfully and displayed significantly lower success rate (RM-2ANOVA, $F_{(3, 7)} = 5.706$, $p = 0.0270$; n = 3 PVJ20- mice, n = 4 PVJ20+ mice). Data represented as mean ± SEM. *, $p < 0.05$; **, $p < 0.01$; ***, $p < 0.001$. Source data are provided as a Source Data file.

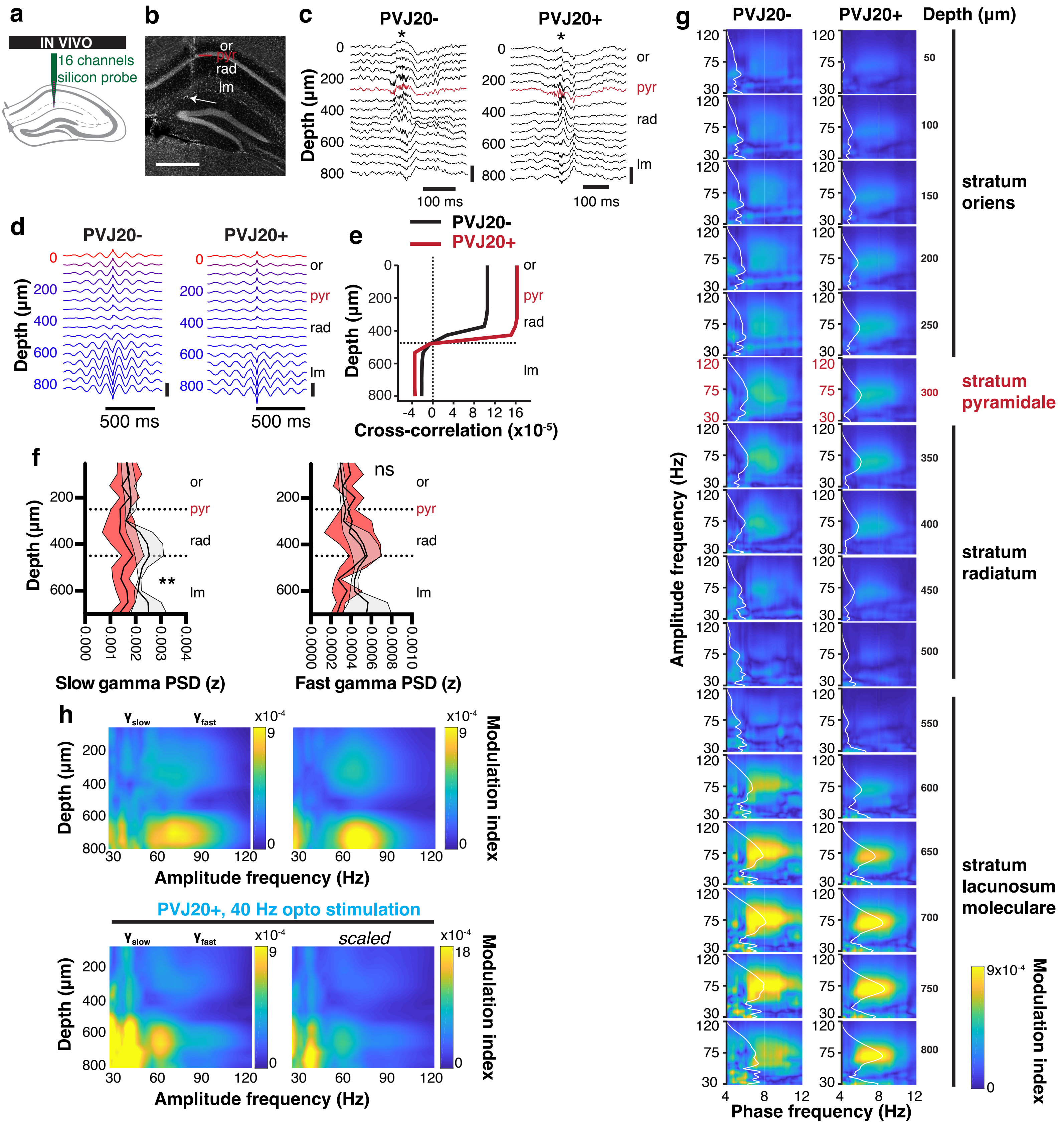

Supplementary figure 3. Transversal phase-amplitude coupling profile. a, recording configuration**. b,** electrode tract and tip location (arrow) in a coronal section of dorsal hippocampus (scale bar: 500 µm). **c,** example CA1 sharp-wave ripples recorded in PVJ20+ mice (right) and their control littermates (left). Star indicates ripple. Putative pyramidal cell layer with strongest ripple power in red (horizontal scale bar: 300 µV). **d**, transversal cross-correlation profile using the first channel (top) as a reference (horizontal scale bar: $2x10^{-4}$ correlation coefficient). **e**, peak cross-correlation across layers (0 indicates phase reversal). **f**, slow (30 - 60 Hz) and fast (60 - 120 Hz) gamma band power spectral densities across layers in PVJ20- (n = 3) and PVJ20+ (n = 3) mice. **g**, comodulograms for each silicon probe recording site. Maximum amplitude frequency coupled to theta (4 - 12 Hz) overlayed (white trace). **h**, maximum amplitude frequency coupled to theta for each recording depth for one example PVJ20- and one example PVJ20+ before (top right panel) and during (bottom panels) 40 Hz optogenetic stimulation. 40 Hz MI is reduced in PVJ20+ in the stratum lm, and restored during 40 Hz opto stimulation (red arrow). Color codes in **f** & **g**: blue, low MI; yellow, high MI. Source data are provided as a Source Data file.

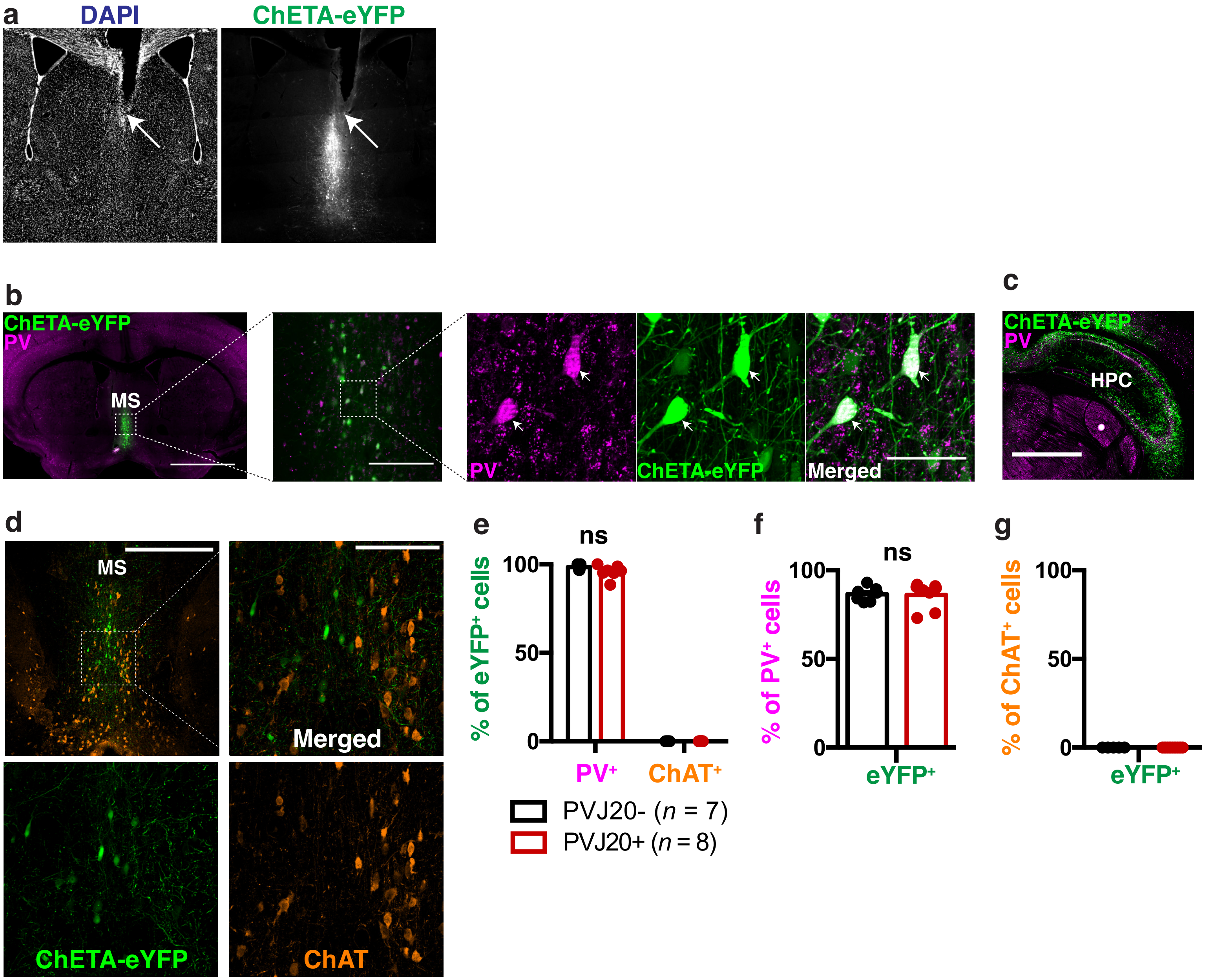

Supplementary figure 4. Histological analysis of medial septum cell transfection. **a**, fiber placement (tip indicated by the arrow) and example transfection. **b**, ChETA-eYFP expression in medial septum parvalbumin neurons (green, eYFP; magenta, PV immunostaining; scalebars: 2 mm (left), 200 µm (center), 50 µm (right). **c**, same but in hippocampus were only fiber tracts were found (scalebar, 500 µm). **d**, immunostaining for choline acetyltransferase (ChAT) and eYFP (scale bars: top left, 500 µm; top right: 100 µm). **e-g**, quantification of specific and non-specific transfection of ChETA-eYFP and eYFP in PVJ20- and PVJ20+ mice. **e**, expression of PV and ChAT did not differ between PVJ20+ and their control PVJ20- littermates ($p = 0.1077$; 2way ANOVA, $F_{(1, 25)} = 2.784$). **f**, expression of eYFP in PV cells did not differ between PVJ20+ (86.06 ± 2.586 %, n = 8) and PVJ20- (86.57 ± 1.518, n = 7; $p = 0.8735$; unpaired t-test, $t_{(13)}=0.1624$). **g**, expression of eYFP in ChAT cells was never observed in PVJ20- nor in PVJ20+ mice. Data expressed as mean ± SEM. ns, not significant. Source data are provided as a Source Data file.

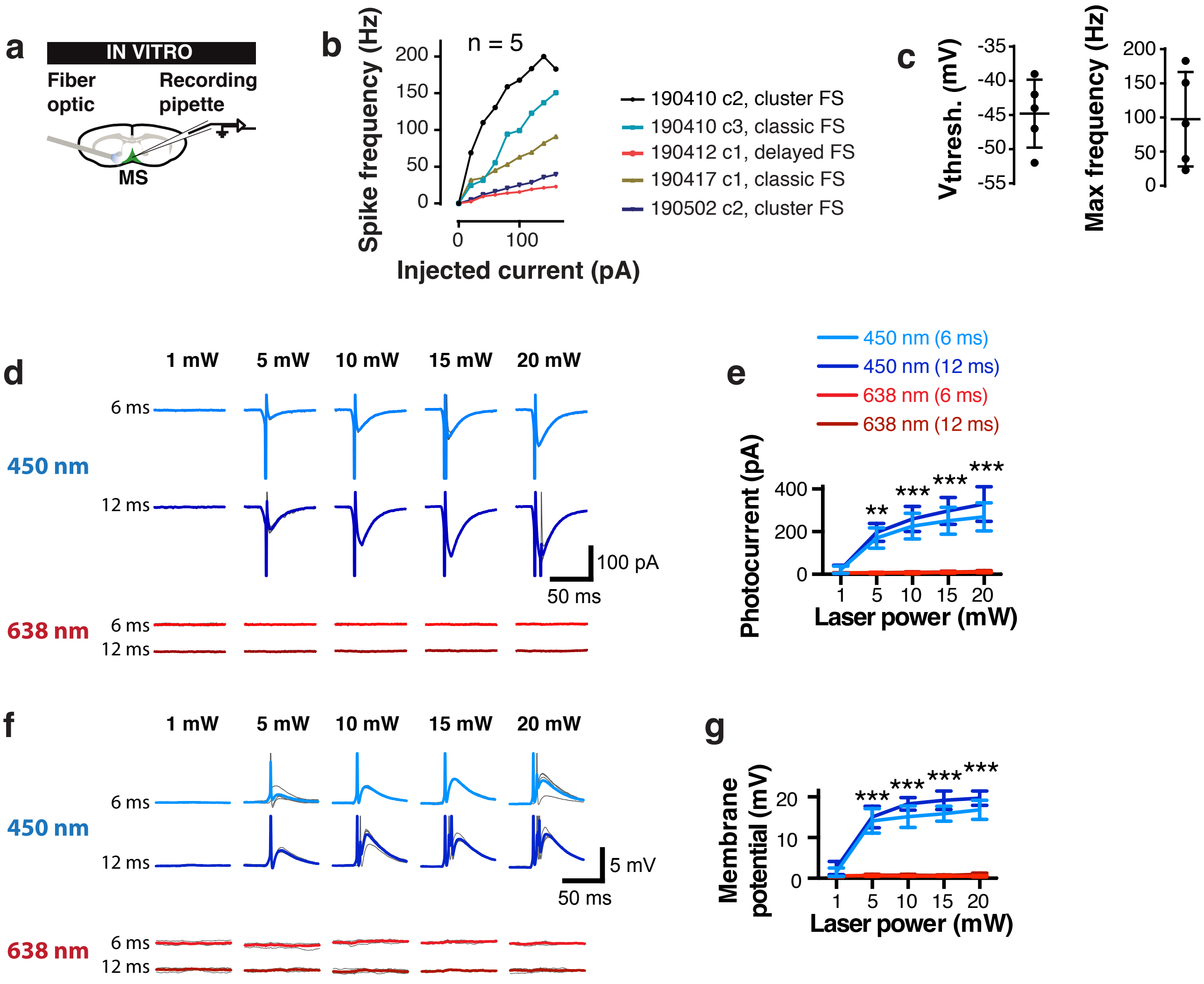

Supplementary figure 5. Optogenetic control of medial septum parvalbumin cells. **a**, in vitro recording configuration. **b**, individual spike frequency responses of visually identified MSPV neurons. **c**, rheobase and max firing frequency. **d**, example responses to varying light stimulation intensities (1 - 20 mW) in voltage clamp. **e**, associated group photocurrents. 450 nm laser stimulation induced significantly larger photocurrents compared to 638 nm stimulation (2ANOVA, $F_{(4, 16)} = 19.78$, $p < 0.0001$; n = 5 neurons). **f**, same as in **d** but in current clamp. **g**, group responses of membrane potential to light stimulation. 450 nm laser stimulation induced significantly larger changes in membrane potential compared to 638 nm stimulation (2ANOVA, $F_{(1, 4)} = 17.13$, $p = 0.0144$). Data expressed as mean ± SEM. ***, 0.001; ****, 0.0001. Source data are provided as a Source Data file.

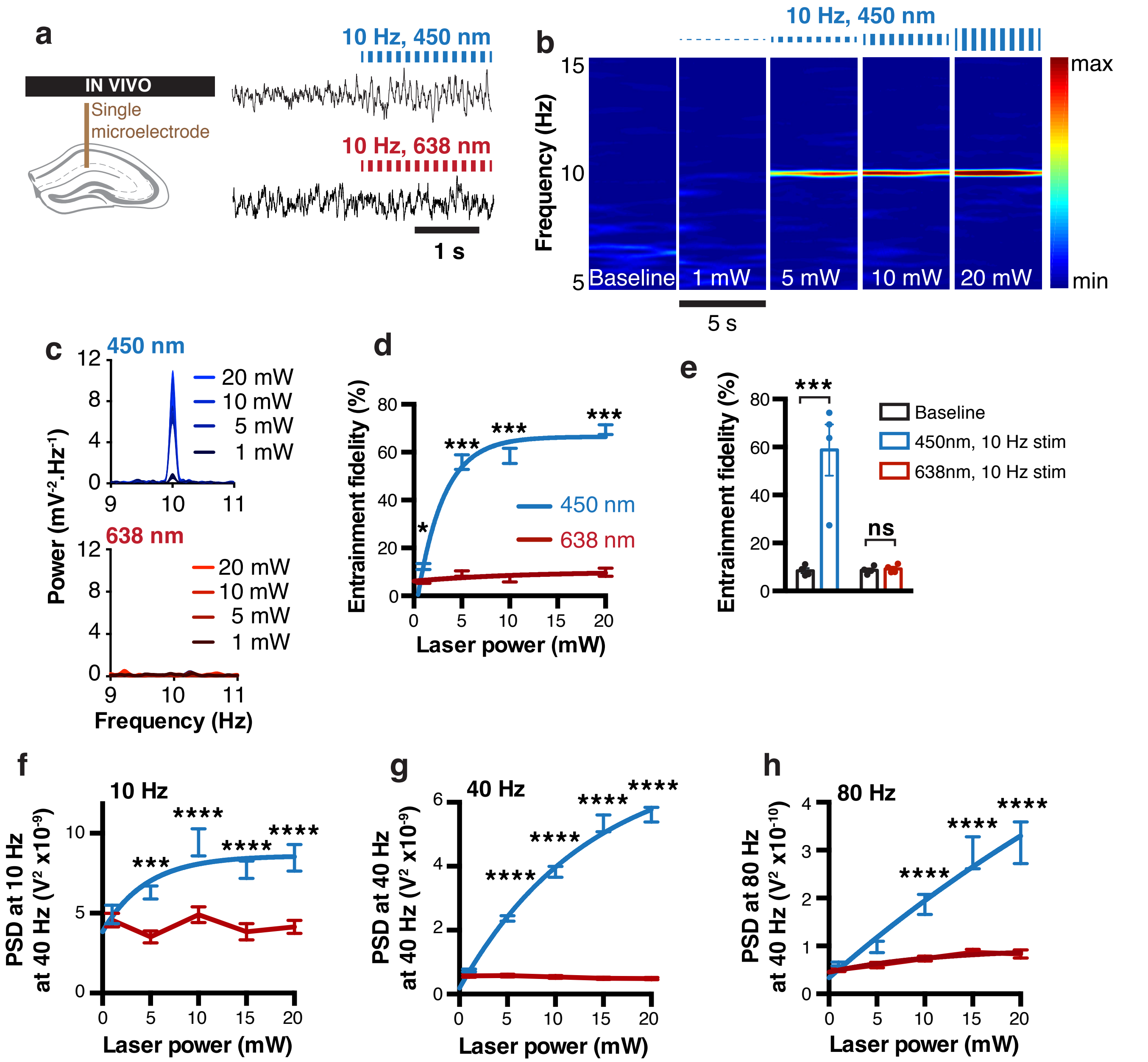

Supplementary figure 6. Input-output responses of CA1 local field potential to laser stimulation of medial septum parvalbumin neurons. a, electrode location (left) and example traces (right) before and during 450 nm (top right) and 638 nm (bottom right) 10 Hz optogenetic stimulation in PVJ20+ mice. **b**, example 5 s spectrograms during optogenetic stimulations at varying laser power. **c**, power spectra in response to increasing laser stimulation intensities at 450 nm (top) and 638 nm (bottom). **d**, entrainment fidelity of theta oscillations in response to 10 Hz stimulations at varying laser intensities is higher at 450 nm (blue line; 2ANOVA, P < 0.0001 for main effect of laser wavelength, *n* = 6 stimulations) compared to 638 nm (red line). **e**, group averages showing large increase in entrainment fidelity using 20 mW, 450 nm optogenetic stimulations at 10 Hz (2ANOVA, P < 0.001 for effect of stim in 450nm condition, Sidak post hoc test). **f**, PSD was directly increased at 10 Hz when stimulated at that frequency (2ANOVA, $F_{(4, 584)} = 6.279$, p < 0.0001, n = 60 stimulations)). **g**, same for 40 Hz (2ANOVA, $F_{(4, 590)} = 117.0$, p < 0.0001, n = 60 stimulations)). **h**, same for 80 Hz (2ANOVA, $F_{(4, 590)} = 21.54$, p < 0.0001, n = 60 stimulations). Data expressed as mean ± SEM. ns, not significant; *, p < 0.05; ***, p < 0.001; ****, p < 0.0001. Source data are provided as a Source Data file.

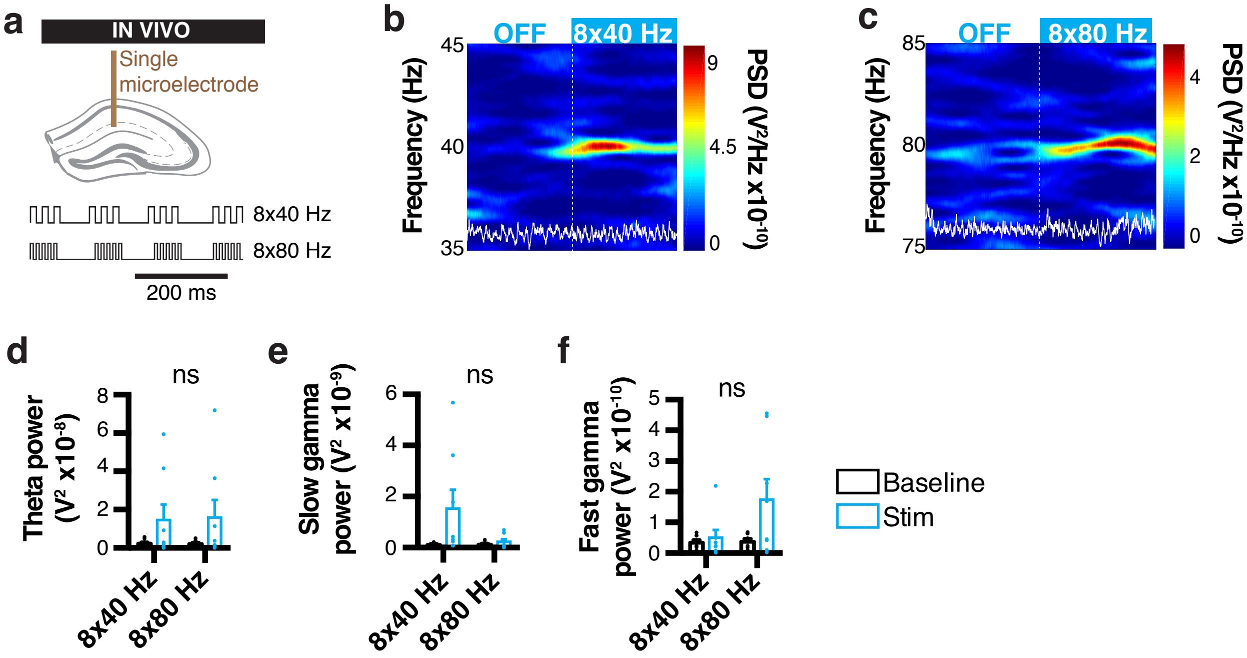

Supplementary figure 7. Optogenetic theta-burst stimulation does not robustly increase gamma power. **a**, recording configuration (top) and stimulation patterns (bottom). **b**, example spectrogram before and during 8x40 Hz theta-burst stimulation. **c**, same using a 8x80 Hz theta-burst stimulation. **d**, effects of optogenetic theta-burst stimulation on theta band power. **e**, effects of optogenetic theta-burst stimulation on slow gamma band power. **f**, effects of optogenetic theta-burst stimulation on fast gamma band power. n = 8 PVJ20+, ChETA mice used in panels **d**-**f**. Data expressed as mean ± SEM. ns, not significant. Test used in **d**-**f**: 2ANOVA. Source data are provided as a Source Data file.

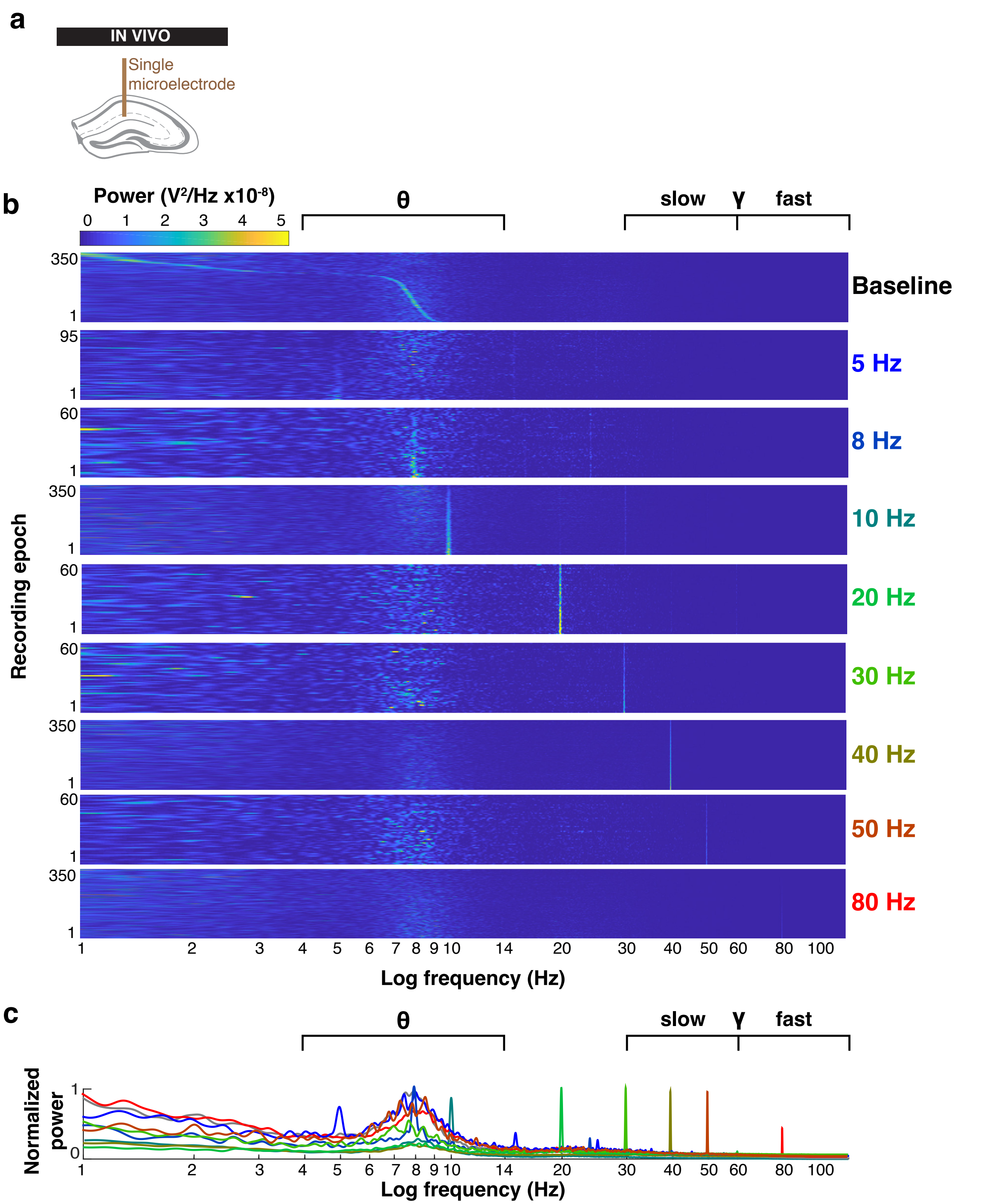

Supplemental figure 8. CA1 local field potential can be robustly entrained at frequencies ranging from 5 to 80 Hz. a, recording configuration. **b**, PSD of each recording epoch (5 s) sorted by the fidelity of the response. **c**, corresponding normalized PSD for each stimulation frequency. Data represented as mean ± SEM (n = 60 stimulations per frequency).

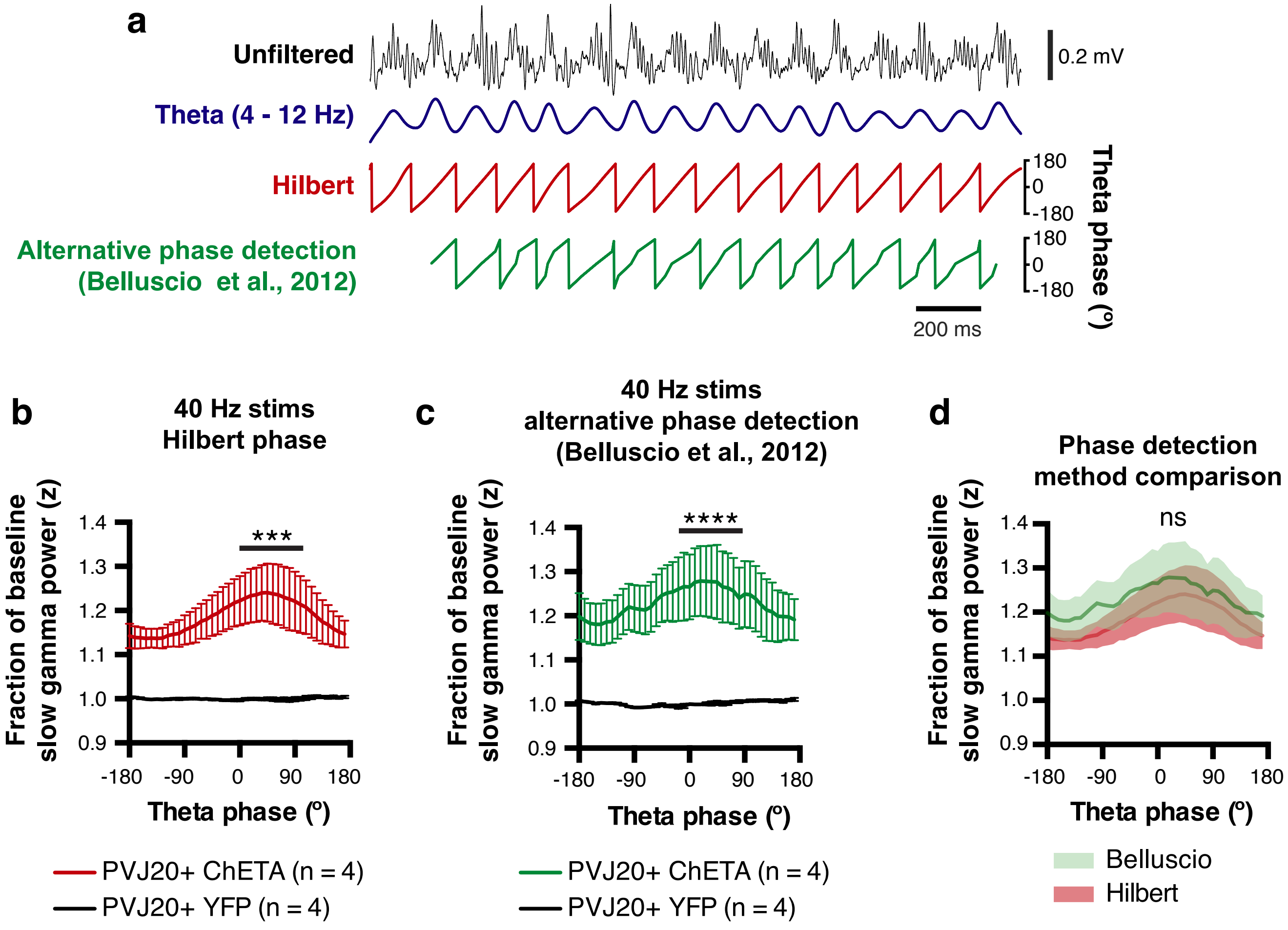

Supplementary figure 9. Control of phase detection during optogenetic stimulation. **a**, example 2 s trace from CA1-LFP with corresponding filtered theta signal (blue), theta phase as derived from the Hilbert transform (red), and theta phase as calculated using the method described in Belluscio et al., 2012 (green). **b**, phase-amplitude coupling computed using Hilbert transformed-derived theta phase (same as in figure 3d). **c**, phase-amplitude coupling computed using theta phase based on the method described in Belluscio et al., 2012. Slow gamma power coupling was significantly increased during 40 Hz stimulation (2ANOVA, $F_{(1, 6)} = 12.91$, $p = 0.0115$ for the main effet of the injected construct; n = 4 PVJ20+, ChETA mice, n = 4 PVJ20+, YFP mice). **d**, comparison of the two different phase extraction methods on the optogenetically induced increase in slow gamma coupling described in figure 3d. No significant difference was found between the two methods (2ANOVA, $F_{(1, 6)} = 0.3220$, $p = 0.5910$, n = 4 PVJ20+, ChETA mice with Belluscio's phase detection method, n = 4 PVJ20+, ChETA mice with Hilbert's phase detection method). Data expressed as mean ± SEM. ns, not significant; ***, $p < 0.001$; ****, $p < 0.0001$. Source data are provided as a Source Data file.

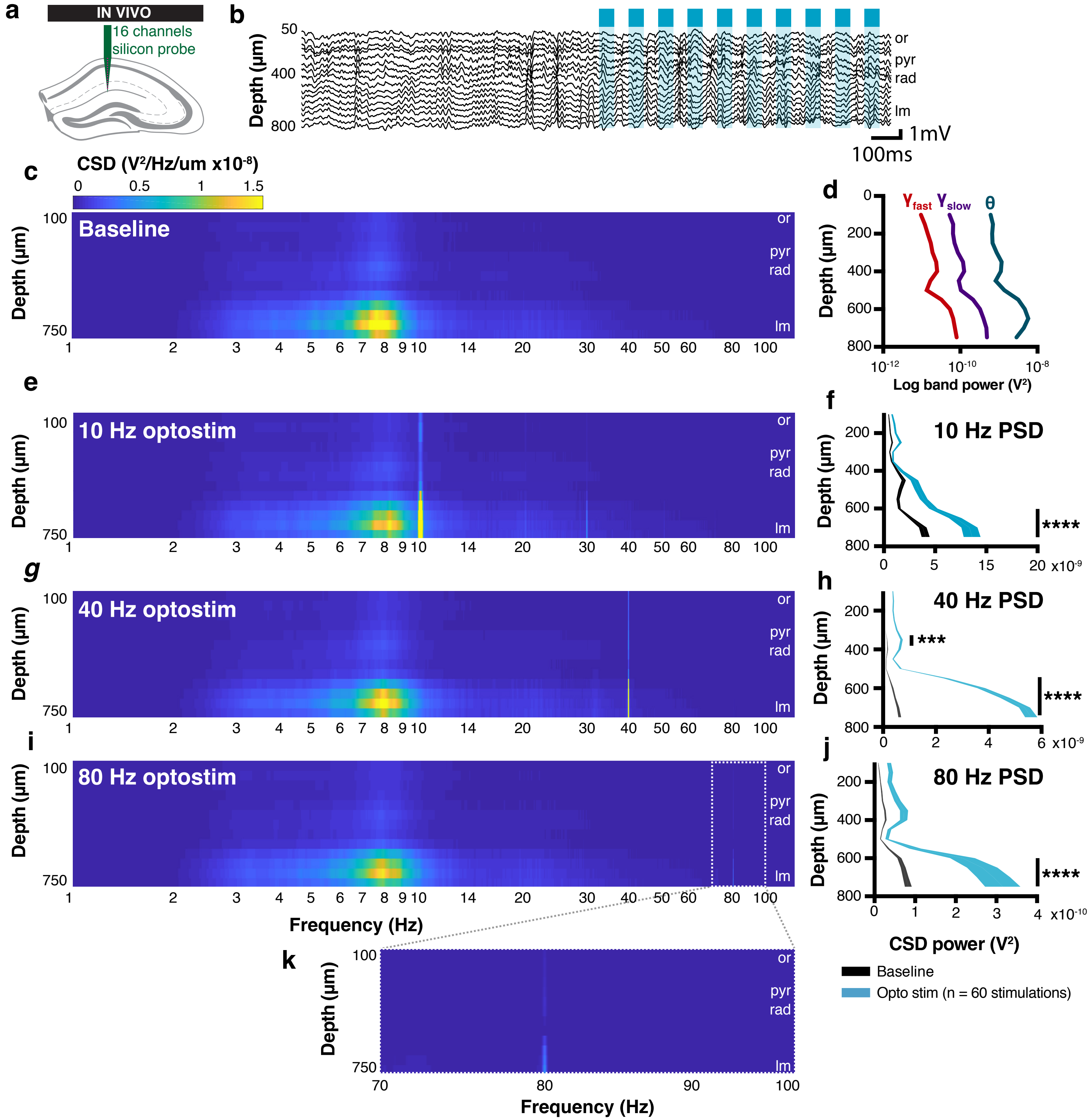

Supplementary figure 10. Effect of stimulation on CA1 PSD transversal profile. a, recording configuration. **b**, example raw LFP traces from a silicon probe recording before and during 10 Hz optogenetic stimulation. **c**, PSD across CA1 layers without stimulation. **d**, theta (blue), slow gamma (purple), and fast gamma band power across CA1 layers without stimulation (calculated from CSD signals). **e**, average transveral profile of PSD during 10 Hz opto stimulation. **f**, corresponding PSD at 10 Hz during stimulation (blue trace) and baseline (black trace). 10 Hz opto simulation significantly increased power in the lm (2ANOVA, $F_{(1, 1568)} = 212.0$, $p < 0.0001$, main effect of stimulation; n = 60 stimulations). **g,** same as **e**, but with 40 Hz. **h**, 40 Hz opto stimulation significantly increased power in the pyr and lm layers (2ANOVA, $F_{(1, 1652)} = 2908$, $p < 0.0001$, main effect of stimulation; n = 6o stimulations). **i**, same as in **e** and **g** for 80 Hz. **j**, 80 Hz opto stimulation significantly increased power in the lm layer (2ANOVA, $F_{(1, 1652)} = 188.4$, $p < 0.0001$, main effect of stimulation; n = 60 stimulations). **k**, enlarged portion of **i**, in the fast gamma band. Data expressed as mean ± SEM. ****, $p < 0.0001$. Source data are provided as a Source Data file.

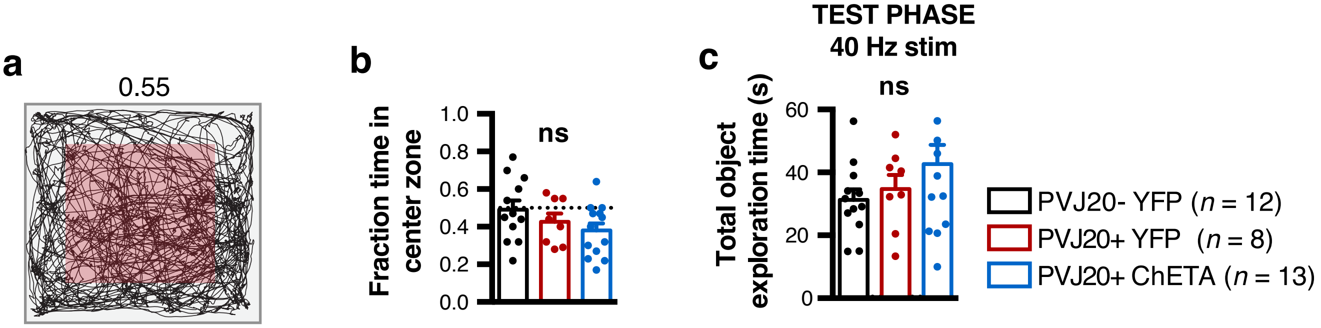

Supplementary figure 11. Baseline thigmotaxis and exploration duration is not different in PVJ20+ mice and not affected by optogenetic stimulation. a, example of mouse trajectory in the open field during habituation (10 min, day 1). Top, corresponding portion of time spent in center zone (red area). **b**, thigmotaxis did not differ between groups (1ANOVA, $F_{(2, 30)} = 1.781$, $p = 0.1857$; n = 12 PVJ20-, YFP mice, n = 8 PVJ20+, YFP mice, and n = 13 PVJ20+, ChETA mice). **c**, total exploration (familiar + displaced) time did not differ between groups (1ANOVA, $F_{(2, 30)} = 1.491$, $p = 0.2414$; n = 12 PVJ20-, YFP mice, n = 8 PVJ20+, YFP mice, and n = 13 PVJ20+, ChETA mice). Data expressed as mean ± SEM. ns, not significant. Source data are provided as a Source Data file.

	Mouse ID								
	**291**	**296**	**299**	**292**	**297**	**301**	**303**	**308**	**312**
**Au1**		*	*	**			*	*	
**AuD**	*	*	*	*	*				
**AuV**				**					
**BLA**	*								
**cc**	**	**	*	***	**	**	**	**	**
**DIEnt**				*			*	*	
**DLEnt**		*					*		
**DS**	*	*							
fi	*	*							
**HPC**	***	***	***	***	***	***	**	***	**
**LPMR**	*								
**LPtA**	*		*		*			*	*
**MPTa**	*		**		*	**		*	**
**RSD**	**	***	**	***	**	**		*	**
**RSGa**			**	**					
**RSGb**	**	**	**	**	**	**	*	**	*
**RSGc**	**	**	**	**	***	**	**	**	**
**S1BF**	*	*	**	**	*	*	*	*	
**S1Tr**	*			**		*			
**S2**	*	*		*					
**TeA**	*	*	*		*		*		*
**V1**	*	*	*	*	**	**		**	*
**V1B**		*	**	**			*	*	*
**V1M**		*	*	**	*			*	*
**V2L**		**	*	**	**	**		*	
**V2ML**	*	**	**	**	*	*	*		*
**V2MM**	**	**	**	**	*	***	*	**	*

Supplementary table 1. Location of plaques in PVJ20+ mice (n = 9). *, at least one plaque observed. **, 1-5 plaques per 50 µm section. ***, >5 plaques per 50 µm section. No star: no plaque was observed. Au1, primary auditory cortex. AuD, secondary auditory cortex, dorsal area. AuV, secondary auditory cortex, ventral area. BLA, basolateral amygdaloid nucleus, anterior part. cc, corpus callosum. DIEnt, dorsal intermediate entorhinal cortex. DLEnt, dorsolateral entorhinal cortex. DS, dorsal subiculum. fi, fimbria of the hippocampus. HPC, hippocampus. LPMR, lateral posterior thalamic nucleus, mediorostral part. LPtA, lateral parietal association cortex. MPtA, medial parietal association cortex. RSD, retrosplenial dysgranular cortex. RSGa, retrosplenial granular cortex, a region. RSGb, retrosplenial granular cortex, b region. RSGc, retrosplenial granular cortex, c region. S1BF, primary somatosensory cortex, barrel field. S1Tr, primary somatosensory cortex, trunk region. S2, secondary somatosensory cortex. TeA, temporal association cortex. V1, primary visual cortex. V1B, primary visual cortex, binocular area. V1M, primary visual cortex, monocular area. V2L, secondary visual cortex, lateral area. V2ML, secondary visual cortex, mediolateral area. V2MM, secondary visual cortex, mediomedial area

		Baseline	Optogenetic stimulation	
		**PVJ20+**	**PVJ20+, 40 Hz**	**PVJ20+, 80 Hz**
**Theta (4-12 Hz)**	**Band power**	**Normal** (fig. 1g,i)	**Normal** (fig. 2k)	**Normal** (fig. 2k)
	**Frequency**	**Normal** (fig. 1f,i)	**Normal** (fig. 4k)	**n/a**
**Slow gamma (30-60 Hz)**	**Band power**	**Decreased** (fig. 1k; 4c)	**Increased** (fig. 2j,l; 3h)	**Unchanged** (fig. 2j,l; 3h)
	**Power at 40 Hz**	**n/a**	**Increased** (fig. 2n)	**n/a**
	**Phase-amplitude coupling**	**Decreased** (fig. 1m; 4e)	**Increased** (fig. 3c,d,i,j)	**Unchanged** (fig. 3e,i)
**Fast gamma (60-120 Hz)**	**Band power**	**Decreased** (fig. 1k; 4d)	**Unchanged** (fig. 2j,m; 3h)	**Unchanged** (fig. 2j,m; 3h)
	**Power at 80 Hz**	**n/a**	**n/a**	**Increased** (fig. 2n)
	**Phase-amplitude coupling**	**Normal** (fig. 1n; 4f)	**Unchanged** (fig. 3f,i)	**Unchanged** (fig. 3g,i)
**Novel object place recognition**		**Decreased** (fig. 4n)	**Increased** (fig. 4n)	**Unchanged** (fig 4n)

Supplementary table 2. Summary of PVJ20+ mice baseline phenotype and effect of optogenetic stimulations. Band power refers to the average amplitude of oscillations in a given frequency band derived from a fast Fourier transform (FFT). Power at 40/80 Hz is obtained by measuring FFT power at a single frequency (40 or 80 Hz). Phase-amplitude coupling is calculated by averageing z-scored slow/fast gamma amplitude values for each theta phase.
