# Supplementary material for: Optogenetic gamma stimulation rescues memory impairments in an Alzheimer’s disease mouse model
Source: Nat Commun. 2019 Nov 22;10:5322. doi: 10.1038/s41467-019-13260-9 (PMC6876640; doi:10.1038/s41467-019-13260-9)
Supplement: Supplementary file 3 — Reporting Summary [file 41467_2019_13260_MOESM3_ESM.pdf]

nature research

Corresponding author(s): Sylvain Williams

Reporting Summary

Nature Research wishes to improve the reproducibility of the work that we publish. This form provides structure for consistency and transparency in reporting. For further information on Nature Research policies, see Authors & Referees and the Editorial Policy Checklist.

Statistical parameters

When statistical analyses are reported, confirm that the following items are present in the relevant location (e.g. figure legend, table legend, main text, or Methods section).

n/a	Confirmed	
☐	☒	The exact sample size (*n*) for each experimental group/condition, given as a discrete number and unit of measurement
☐	☒	An indication of whether measurements were taken from distinct samples or whether the same sample was measured repeatedly
☐	☒	The statistical test(s) used AND whether they are one- or two-sided<br>*Only common tests should be described solely by name; describe more complex techniques in the Methods section.*
☒	☐	A description of all covariates tested
☐	☒	A description of any assumptions or corrections, such as tests of normality and adjustment for multiple comparisons
☐	☒	A full description of the statistics including central tendency (e.g. means) or other basic estimates (e.g. regression coefficient) AND variation (e.g. standard deviation) or associated estimates of uncertainty (e.g. confidence intervals)
☐	☒	For null hypothesis testing, the test statistic (e.g. *F*, *t*, *r*) with confidence intervals, effect sizes, degrees of freedom and *P* value noted<br>*Give P values as exact values whenever suitable.*
☒	☐	For Bayesian analysis, information on the choice of priors and Markov chain Monte Carlo settings
☒	☐	For hierarchical and complex designs, identification of the appropriate level for tests and full reporting of outcomes
☐	☒	Estimates of effect sizes (e.g. Cohen's *d*, Pearson's *r*), indicating how they were calculated
☐	☒	Clearly defined error bars<br>*State explicitly what error bars represent (e.g. SD, SE, CI)*

Our web collection on statistics for biologists may be useful.

Software and code

Policy information about availability of computer code

Data collection	All electrophysiological data was collected on Cheetah (Neuralynx, USA)
Data analysis	All data was analysed with custom writen Matlab code available upon request

For manuscripts utilizing custom algorithms or software that are central to the research but not yet described in published literature, software must be made available to editors/reviewers upon request. We strongly encourage code deposition in a community repository (e.g. GitHub). See the Nature Research guidelines for submitting code & software for further information.

Data

Policy information about availability of data

All manuscripts must include a data availability statement. This statement should provide the following information, where applicable:

- Accession codes, unique identifiers, or web links for publicly available datasets
- A list of figures that have associated raw data
- A description of any restrictions on data availability

All data generated or analysed during this study are either included in this published article or are available from the corresponding authors on reasonable request

Field-specific reporting

Please select the best fit for your research. If you are not sure, read the appropriate sections before making your selection.

☒ Life sciences ☐ Behavioural & social sciences ☐ Ecological, evolutionary & environmental sciences

For a reference copy of the document with all sections, see nature.com/authors/policies/ReportingSummary-flat.pdf

Life sciences study design

All studies must disclose on these points even when the disclosure is negative.

Sample size	Our initial study (fig 1.) with silicon probes (n = 3 PVJ20+, n = 3 PVJ20-) allowed us to examine transversal profile of hippocampal oscillations. While these are small groups, we replicated these recordings in the lacunosum moleculare using larger groups (n = 10 PVJ20+, n = 10 PVJ20-). In our in vitro study, ChETA transfected neuron always responded to 450 nm light and never responded to 638nm light (n = 5), which was sufficient to perform statistical analyses. We tested different stimulation patterns on n = 24 mice in vivo (fig 2), and found consistent results over the course of severals batches (see bellow). In experiments with optogenetic stimulations (fig 3), only ChETA transfected (but not YFP controls) displayed responses to laser stimulations, so n = 4 ChETA and n = 4 YFP controls were sufficient for statistical significance. In our behavioural analyses (fig 4), we performed 2way, repeated measure ANOVA on n = 31 mice, and computed partial eta-squared as a measure of effect size, which was considered of high magnitude (0.26).
Data exclusions	n = 1 mouse displayed dental occlusion, subsequent malnutrition, and thus was excluded from the study after surgical implantation
Replication	5 batches containing homogenous groups (for genotype, treatment, sex) were used for the initial electrophysiological characterization in figure 1 and replicated similar results consistently over the course of 3 years. 4 batches of mice were used to establish the effects of optogenetic stimulations on behavior (figure 3) over the course of two year and both batches replicated comparable results.
Randomization	After determining genotypes by PCR, J20+ and J20- mice were randomly assigned a treatment condition so that groups were balanced for sex, genotype and treatment
Blinding	Experimenters and analysts were blind to the subject genotype (and treatment when involved)

Reporting for specific materials, systems and methods

Materials & experimental systems

n/a	Involved in the study	
☒	☐	Unique biological materials
☐	☒	Antibodies
☒	☐	Eukaryotic cell lines
☒	☐	Palaeontology
☐	☒	Animals and other organisms
☒	☐	Human research participants

Methods

n/a	Involved in the study	
☒	☐	ChIP-seq
☒	☐	Flow cytometry
☒	☐	MRI-based neuroimaging

Antibodies

Antibodies used	anti-Parvalbumin, monoclonal IgG1 clone PARV-19 produced in mouse (Sigma-Aldrich, catalog number: P3088)<br>anti-GFP produced in rabbit, (Life Technologies, catalog numberL A-11122)<br>anti-Choline actyltransferase produced in goat (Millipore, AB144P)<br>goat anti-rabbit coupled to Alex488 (Molecular Probes A11034)<br>goat anti-mouse IgG1 coupled to Alexa 555 (Life Technologies, A21127)<br>donkey anti-goat coupled to Alexa 647 (Jackson Immunoresearch, 705-605-147)<br>donkey anti-chicken coupled to Alexa 488 (Jackson Immunoresearch, 703-545-155)
Validation	Primary antibodies were controled for non-specific staining by incubating secondary antibodies without initial primary antibody incubation. Anti-parvalbumin and anti-chat were further cross-validated by performing the staining in PV-Tom mice (PVCre mouse crossed with a tdTomato reporter mouse) and ChAT-Tom mice, respectively. Anti-GFP was further cross-validated by performing the staining in PVChR2-YFP mice (PVCre mouse crossed with a ChR2-eYFP knock-in mouse line that expresses eYFP in PV cells)

Animals and other organisms

Policy information about studies involving animals; ARRIVE guidelines recommended for reporting animal research

Laboratory animals	Male mice expressing a mutant form of the human amyloid protein precursor bearing both the Swedish (K670N/M671L) and the Indiana (V717F) mutations (APPSwInd) under the control of the PDGFB promoter [B6. Cg-Tg(PDGFB-APPSwInd), (Jackson laboratory)] were bred with female parvalbumin-cre mice (Jackson Laboratory). This mouse line is termed PVJ20 throughout the current study, and both male and female PVJ20 specimen were used throughout this study.
Wild animals	The study did not involve wild animals
Field-collected samples	The study did not involve samples collected from the field
